# The role of the SIRT1-BMAL1 pathway in regulating oxidative stress in the early development of ischaemic stroke

**DOI:** 10.1038/s41598-024-52120-5

**Published:** 2024-01-20

**Authors:** Jing Shi, Weirong Li, Xiaobo Ding, Feng Zhou, Chenxi Hao, Miao He, Fan Wang, Xinyi Li

**Affiliations:** 1 Department of Neurology, Third Hospital of Shanxi Medical University, Shanxi Bethune Hospital, Shanxi Academy of Medical Sciences Tongji Shanxi Hospital, Taiyuan, China; 2 The Ninth Clinical Medical College Affiliated with Shanxi Medical University, Taiyuan, China; 3 Cardiovascular Hospital Affiliated to Shanxi Medical University, Taiyuan, China; 4 School of Public Health, Shanxi Medical University, Taiyuan, China; 5 The First Clinical Medical College Affiliated with Shanxi Medical University, Taiyuan, China; 6 Department of Neurology, Aerospace Center Hospital, Peking University Aerospace Clinic College of Medicine, Beijing, China; 7 Tongji Hospital, Tongji Medical College, Huazhong University of Science and Technology, Wuhan, China

**Keywords:** Cerebrovascular disorders, Stroke

## Abstract

Oxidative stress is the primary cause of ischaemic stroke and is closely related to circadian rhythm. However, the mechanism by which circadian rhythm regulates oxidative stress in ischaemic stroke remains elusive. The Silent Information Regulator 1 (SIRT1) controls circadian rhythm by activating the transcription of the circadian clock core protein Basic Helix-Loop-Helix ARNT Like 1 (BMAL1) through deacetylation. Studies have shown that the SIRT1-BMAL1 pathway can regulate oxidative stress. To investigate its correlation with oxidative stress, we examined the expression levels and influencing factors of SIRT1-BMAL1 at different times in ischaemic stroke patients and analyzed their clinical indexes, oxidative stress, and inflammatory factor indicators. The expression levels of oxidative stress and inflammatory factor indicators, including malondialdehyde (MDA), superoxide dismutase (SOD), interleukin-6 (IL-6), and tumor necrosis factor-a (TNF-α), SIRT1, and BMAL1, were detected in ischaemic stroke patients within 4.5 h of onset and in non-stroke patients. Patients were divided into four subgroups based on onset time: subgroup 1 (0:00–05:59); subgroup 2 (06:00–11:59); subgroup 3 (12:00–17: 59); and subgroup 4 (18:00–23:59). Our results showed higher MDA, IL-6, and TNF-α levels, and lower SOD, SIRT1, and BMAL1 levels in ischaemic stroke patients compared to control patients (*P* < 0.05). Among the four subgroups, the content of MDA, IL-6, and TNF-α was highest in patients with ischaemic stroke onset from subgroup 2 (06:00–11:59), while the expression levels of SOD, BMAL1, and SIRT1 were lowest in patients with ischaemic stroke in subgroup 2. Additionally, myeloperoxidase (MPO) reached the highest value showing the same trends consistent with MDA, IL-6, and TNF-ɑ and opposite trends consistent with SOD, BMAL1, and SIRT1. However, triglycerides (TGs), total cholesterol (TC), low-density lipoprotein (LDL), high-density lipoprotein (HDL), immediate blood glucose, immediate diastolic blood pressure, immediate systolic blood pressure, and homocysteine (HCY) did not show any statistically significant circadian rhythm changes (*P* > 0.05). Our findings suggest that the SIRT1-BMAL1 pathway may be involved in early oxidative stress in ischaemic stroke, which may be related to MPO.

## Introduction

Ischaemic stroke is one of the leading causes of mortality and disability globally, with ischaemic stroke being the primary cause of disability-adjusted life years (DALYs) and the third leading cause of death In China^1,2^. Intravenous thrombolysis and endovascular therapy are the most effective rescue measures, but time constraints limit their efficacy for more than half of patients. Thus, investigating the early mechanisms of ischemic stroke development is crucial. Reactive oxygen species (ROS) are generated after an ischaemic stroke results in oxidative stress and brain damage in nerve cells^3^. As nerve cells have a high metabolism and weak antioxidant capacities^4,5^, reducing oxidative damage in the early stages of ischaemic stroke is an established intervention strategy^6^. However, the precise mechanism behind oxidative stress in ischaemic neurons remains unclear.

The incidence of ischaemic stroke peaks in the early morning, and ischaemic strokes occurring at this time have a higher mortality rate after adjusting for parameters such as age, sex, and severity^7^. Studies on mice suggest that compared to other time points, the occurrence of acute ischemia–reperfusion injury including the cerebral infarct volume, brain swelling degree, neurological deficit score, neuronal survival, and apoptosis is improved when it happens at midnight, which may be attributed to the circadian rhythm^8^. The circadian rhythm results from periodic changes in protein concentration in cells. The oscillatory changes in these proteins’ abundance, known as circadian clock proteins, regulate physiological functions consistent with the light–dark cycle^9^. One key protein involved in the formation of the circadian rhythm is the Basic Helix-Loop-Helix ARNT Like 1 (BMAL1) protein, which regulates target gene expression via feedback loops involving transcription and translation oscillations. BMAL1 plays a crucial role in forming the circadian rhythm. Animal studies have shown that BMAL1 is also involved in oxidative stress in ischaemic stroke. Knockout of the BMAL1 gene resulted in decreased cellular defenses against oxidative damage^10^. The brain-specific deletion of Bmal1 leads to significant activation of astrocytes, as well as the occurrence of neuronal oxidative damage and impaired expression of multiple redox defense genes^11^. Exacerbate the oxidative damage to neurons. Overexpression of BMAL1 could activate hypoxia-inducible factor (HIF)-1α, indicating a protective role in neurons by regulating oxidative stress^12^.

As an NAD + -dependent sirtuin, the silent information regulator 1 (SIRT1) controls circadian rhythms by activating BMAL1 transcription and is known as an enzymatic regulator of the circadian rhythms^13^. SIRT1 is a widely expressed protein with a complex role in regulating oxidative stress and inflammation^14–16^. The SIRT1-BMAL1 pathway also plays an important role in ischaemic stroke^8,17–19^. A study found that hypoxia increased the mRNA level of PGC-1α in wild-type mice, and increased PGC-1α expression reduced oxidative stress-mediated neuronal death. SIRT1 can directly affect the activity of PGC-1α through deacetylation, thus protecting neurons^20^. Studies have shown that inhibiting oxidative stress induced by the SIRT1 signaling pathway in vivo and in vitro can alleviate reperfusion injury after stroke^21^. BMAL1 has a protective effect on neuronal apoptosis after stroke^22^. Therefore, this study aimed to evaluate the expression changes of the SIRT1-BMAL1 pathway in regulating early oxidative stress response in ischaemic stroke and proposes a reference strategy to effectively restore oxidation–reduction balance and reduce early neuronal damage caused by ischaemic stroke.

## Materials and methods

This prospective study was conducted between June 2021 to June 2023 at the Department of Neurology in Taiyuan Central Hospital and Shanxi Cardiovascular and Cerebrovascular Hospital, located in Shanxi Province. The study was approved by the ethics committee in 2021 (No: 2021019) according to the Declaration of Helsinki, and all participants provided written informed consent.

### General information

The study included patients aged 18–80 with the acute ischaemic cerebrovascular disease within 4.5 h of onset, as defined in the 2018 European Guidelines for Diagnosis and Treatment of Acute Ischaemic Stroke. Exclusion criteria were cerebral hemorrhage or subarachnoid hemorrhage, cardiogenic cerebral embolism, cerebral apoplexy due to hematological diseases, tumors, etc., serious complications such as liver, kidney, hematopoietic, endocrine, and osteoarthritis diseases, mental disorder or severe dementia, use of lipid-lowering and plaque-stabilizing drugs, hormone drugs, and immunosuppressants in the past 3 months, recent chronic or active infection, and other serious life-threatening diseases with an expected survival period < 3 months. Patients information was collected, including age, sex, smoking history, drinking history, hypertension history, diabetes history, atrial fibrillation history, and laboratory indicators at admission such as blood glucose, blood pressure, total cholesterol, triglycerides (TGs), high-density lipoprotein (HDL) cholesterol, low-density lipoprotein (LDL) cholesterol, homocysteine (HCY), and myeloperoxidase (MPO). Additionally, patients were divided into four subgroups based on stroke onset time: subgroup 1 (0:00–05:59); subgroup 2 (06:00–11:59); subgroup 3 (12:00–17:59); and subgroup 4 (18:00–23:59). The blood collection time in all subgroups was within the specified time limit.

To control for confounding factors, the control group was comprised of individuals with peripheral vestibular vertigo or cervical spondylosis who were matched in terms of age and sex to the study group. The control group had no symptoms or history of ischaemic stroke or other neurodegenerative disorders and was tested using the same detection method at the same time point as the study groups. Additionally, all enrolled patients had no previous record of sleep disturbances or recent travel across different time zones.

### NIHSS score

The neurological deficit score of the study group upon admission was assessed using the National Institutes of Health Stroke Scale (NIHSS), with scores evaluated by two skilled professionals trained in its use.

### Detection of the oxidative stress factors including malondialdehyde (MDA) and superoxide dismutase (SOD), inflammatory factors including interleukin-6 (IL-6) and tumor necrosis factor-α (TNF-α), SIRT1, and BMAL1.

The study initially collected 5 mL of peripheral venous blood from all participants in an EDTA anticoagulant tube before any clinical intervention. Mononuclear cells were isolated under an ultraclean workbench within one hour using a protocol that involved adding 1 volume of fresh whole blood to 3 volumes of erythrocyte lysate, followed by centrifugation at 450 × g for 10 min at 4 °C to pellet leukocytes. Twice the volume of erythrocyte lysate was added, and the mixture was centrifuged again to obtain the leukocyte pellet. After discarding the supernatant, isolated cells were placed in a 1.5 mL EP tube and stored at -80 °C until ELISA and Western blot detection were performed.

#### The expression levels of oxidative stress factors (e.g., MDA and SOD) and inflammatory factors (e.g., IL-6 and TNF-α) were measured by ELISA

The MDA, SOD, IL-6, and TNF-α contents in the samples were analyzed using the human MDA ELISA kit, human SOD ELISA kit, human IL-6 ELISA kit, and human TNF-α ELISA kit by the competition method. Samples were added to the enzyme-labeled wells that were pre-coated with antibodies. Then, biotin-labeled recognition antigens were added and incubated at 37 °C for 30 min. The antigen and solid-phase antibody competed for binding, forming an immune complex. After washing with PBST to remove unbound biotin antigen, avidin-HRP was added and incubated at 37 °C for 30 min. Once avidin-HRP combined fully with the biotin antigen, it was washed, and color-developing solutions A and B were added. The mixture was incubated at 37 °C for 10 min to develop the color. Finally, the reaction was terminated by adding the stop solution. The OD value was read and calculated within 10 min^23^.

#### Western blot to detect the expression levels of SIRT1 and BMAL1

The isolated monocytes were homogenized with liquid nitrogen, followed by the addition of 1 ml of PBS and centrifuged at 500 × g for 5 min. The cells were then briefly placed in an ice bath at maximum power (3 × 10 s), and the resulting supernatant was collected by centrifugation at 12,000 rpm for 15 min at 4 °C. The protein concentration was determined using the BCA protein quantification method and stored at −20 °C for later use. The SDS‒PAGE gel electrophoresis was performed, and the resulting protein bands were transferred to a PVDF membrane via transfer electrophoresis. After blocking the membrane for 1 h at room temperature, the primary antibody incubation was carried out overnight at 4 °C. The membrane was subsequently washed with 1 × TBST and incubated with a secondary antibody for 60 min. After washing the membrane to remove free secondary antibodies, an ECL kit was used to visualize the results. The membrane was imaged using a chemiluminescence imaging system. The gray value of WB strip is analyzed using ImageJ software.

### Statistical analysis

The data were analyzed using SPSS 25.0 and R 4.2.0 statistical software. Graph Pad Prism 8.0 software was utilized for graphing, while Image J2x software was employed for analyzing the gray value of all Western Blot bands. Following a normality test, the measurement data that conformed to a normal distribution were expressed as "X^−^(mean) ± S(SD)". Two independent sample t-tests compared means between two groups. Skewed distribution data was represented by M(P25, P75) and compared using z tests. One-way ANOVA was used for comparisons among multiple groups, with a homogeneity of variance test performed. Count data were expressed as frequencies and percentages (n, %) and analyzed using the χ2 test. Correlation analyses between BMAL1, SIRT1, IL-6, TNF-α, MDA, and SOD were performed by Pearson correlation analysis. The test level was two-sided α = 0.05, and *P* < 0.05 was considered statistically significant. Western blot strips were analyzed by ImageJ 2 × software for grey value analysis.

### Ethical approval

The study has been approved by the Medical Ethics Committee of Taiyuan Central Hospital (Approval No: 2021019). We assure that this study strictly adheres to the principles of the 1964 Helsinki Declaration and its subsequent revisions. Throughout the research process, we have followed medical ethics principles and willingly complied with relevant national laws and regulations. We respect the autonomy of the participants and adhere to the principles of beneficence, non-maleficence, and justice. The project is being conducted according to the approved protocol by the ethics committee, and informed consent has been obtained from the participants or their authorized representatives. Comprehensive procedures for informed consent have been implemented to effectively protect the rights and safety of the participants.

## Results

### General data analysis of the study group and the control group

Table 1 presents the general information on the study and control groups. The research group included 100 participants, comprising 63 males and 37 females, with an average age was 65.45 ± 6.54 years. Among them, 73 patients had anterior circulation infarction and 27 patients had posterior circulation infarction. The control group consisted of 100 individuals, including 56 males and 44 females, with an average age was 64.33 ± 10.65 years. Among all the participants, 126 were from Taiyuan Central Hospital and 74 were from Shanxi Cardiovascular and Cerebrovascular Disease Hospital. Baseline levels, such as hypertension, diabetes, coronary heart disease, smoking history, drinking history, and atrial fibrillation history, did not significantly differ between the two groups (P > 0.05). There were no significant differences in TG, TC, LDL, HDL, HCY, and immediate diastolic blood pressure (*P* > 0.05) when comparing laboratory test indicators between the two groups. However, there were statistically significant differences (*P* < 0.05) in MPO, immediate blood sugar, and immediate systolic blood pressure between the study groups and the control group, with the study group having higher levels.

**Table 1:** General data of the study group and the control group.

Item	Study group (n = 100)	Control group (n = 100)	t/z/ × 2	*P* value
Age	65.45 ± 6.54	64.33 ± 10.65	0.897	0.371
Gender (M/F)	63/37	56/44	1.017	0.313
History of hypertension (Y/N)	55/45	50/50	0.501	0.479
History of diabetes (Y/N)	54/46	51/49	0.180	0.671
History of coronary heart disease (Y/N)	16/84	11/89	1.070	0.301
Smoking history (Y/N)	53/47	43/57	2.003	0.157
Alcohol history (Y/N)	28/72	33/67	0.590	0.443
Atrial fibrillation (Y/N)	10/90	13/87	0.442	0.506
TG (mmol/l, M (P25, P75))	1.63 (1.23, 2.41)	1.52 (1.14, 2.08)	-1.767	0.077
TC (mmol/l, X ± S)	4.31 ± 0.81	4.44 ± 0.96	−1.062	0.289
LDL (mmol/l, M (P25, P75))	2.71 (2.19, 3.05)	2.60 (1.82, 3.56)	−0.367	0.714
HDL (mmol/l, M (P25, P75))	0.89 (0.79, 1.03)	1.00 (0.77, 1.20)	−1.793	0.073
HCY (μ mol/l, M (P25, P75))	18.10 (14.73, 22.28)	16.80 (14.20, 19.40)	−1.660	0.097
MPO (mmol/l, M (P25, P75))	253.03 ± 131.89	137.07 ± 23.10	8.660	< 0.001
Immediate blood glucose (mmol/l, M (P25, P75))	6.8 (6.0, 8.3)	5.1 (4.6, 6.5)	−7.181	< 0.001
Immediate systolic blood pressure (mmHg, M (P25, P75))	136 (128, 149)	132 (118, 149)	−2.262	0.024
Immediate diastolic pressure (mmHg, M (P25, P75))	80 (74, 88)	78 (70, 90)	−1.325	0.185

### Comparison of laboratory indicators and NIHSS scores of four subgroups in the study group

The research group was stratified into four subgroups (subgroup 1: 0:00–05:59, subgroup 2: 06:00–11:59, subgroup 3: 12:00–17:59, and subgroup 4: 18:00–23:59, 25 people per group) based on the stroke onset time. Analysis of laboratory indicators and NIHSS scores immediately after admission showed no significant circadian rhythm changes in TGs, TC, LDL, HDL, immediate blood glucose, immediate diastolic blood pressure, immediate systolic blood pressure, and HCY among the four subgroups (*P* > 0.05). However, statistically significant differences were observed in MPO and NIHSS scores among the four groups, indicating a significant circadian rhythm (*P* < 0.05). In particular, MPO values were highest in ischemic stroke patients with onset at subgroup 2 (6:00–11:59) and lowest in those with onset at subgroup 4 (18:00–23:59). Conversely, the NIHSS scores showed rhythmic change inconsistent with MPO, being highest in ischemic stroke patients with onset at subgroup 1 (00:00–05:59) and lowest in those with onset at subgroup 3 (12:00–17:59) (Table 2).

**Table 2:** Laboratory metrics and NIHSS scores among the four subgroups.

Item	Subgroups 1	Subgroups 2	Subgroups 3	Subgroups 4	F	*p*
Age (year, x ± s)	65.48 ± 7.30	64.40 ± 6.00	64.16 ± 6.47	67.76 ± 6.05	1.610	0.192
Male (case, %)	14 (56.0%)	18 (72.0%)	16 (64.0%)	15 (60.0%)	1.502	0.682
History of hypertension (case, %)	12 (48.0%)	17 (68.0%)	14 (56.0%)	12 (48.0%)	2.707	0.439
History of diabetes (case, %)	11 (44.0%)	16 (64.0%)	14 (56.0%)	13 (52.0%)	2.093	0.553
History of coronary heart disease (case, %)	6 (24.0%)	3 (12.0%)	6 (24.0%)	1 (4.0%)	5.467	0.141
Atrial fibrillation (case, %)	3 (12.0%)	2 (8.0%)	0 (0.0%)	5 (20.0%)	5.755	0.133
Smoking history (case, %)	15 (60.0%)	14 (56.0%)	13 (52.0%)	11 (44.0%)	1.405	0.704
Alcohol history (case, %)	6 (24.0%)	9 (36.0%)	9 (36.0%)	4 (16.0%)	3.571	0.312
TG (mmol/L, M (P25, P75))	1.57 (1.18, 2.55)	1.67 (1.16, 2.27)	1.55 (1.23, 2.19)	1.87 (1.29, 2.45)	1.296	0.730
TC (mmol/l, x ± s)	4.35 ± 0.62	4.13 ± 1.03	4.31 ± 0.79	4.44 ± 0.75	0.622	0.602
LDL (mmol/L, M (P25, P75))	2.93 (2.37, 3.16)	2.34 (1.91, 2.78)	2.59 (2.14, 3.50)	2.82 (2.35, 2.97)	6.078	0.108
HDL (mmol/L, M (P25, P75))	0.89 (0.84, 0.98)	0.86 (0.69, 1.02)	0.93 (0.83, 1.06)	0.88 (0.79, 1.09)	2.857	0.414
Immediate blood glucose (mmol/L, M (P25, P75))	6.7 (6.0, 7.7)	6.80 (6.00, 9.55)	7.0 (6.4, 8.4)	6.7 (5.6, 8.4)	2.096	0.553
Immediate systolic blood pressure (mmHg, x ± s)	135 ± 11	142 ± 15	136 ± 10	138 ± 11	1.607	0.199
Immediate diastolicblood pressure (mmHg, x ± s)	78 ± 8	83 ± 11	80 ± 8	82 ± 8	1.28	0.286
HCY (μ mol/l, M (P25, P75))	18.60 (13.75, 22.20)	18.40 (15.85, 21.05)	18.50 (14.30, 22.75)	17.40 (11.45, 22.45)	1.208	0.751
MPO (ng/mL, M (P25, P75))	241.46 ± 144.14	323.43 ± 107.05	267.16 ± 137.64	170.31 ± 60.81	25.505	< 0.01
NIHSS (score, M (P25, P75))	7 (5, 10)	5 (3, 7)	3 (2, 4)	4 (3, 5)	32.072	< 0.001

### MDA, SOD, IL-6, TNF-α, BMAL1, and SIRT1 in the study group and control group

**Figure 1:**
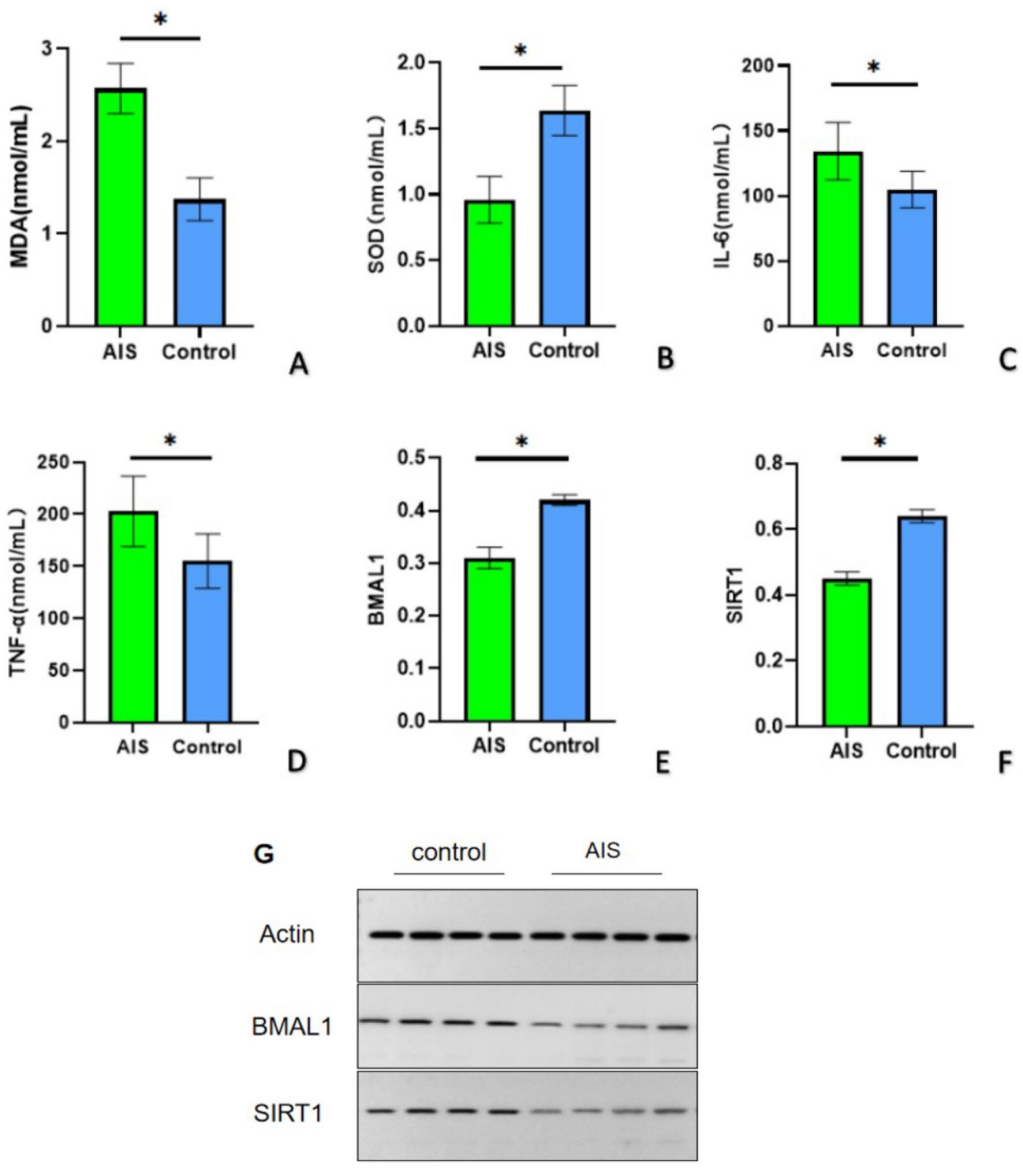
Comparison of MDA, SOD, IL-6, TNF-α, BMAL1 and SIRT1 between the study and control groups. (**A**) The MDA content in the study group was significantly higher than in the control group. (**B**) The SOD content in the study group was significantly lower than in the control group. (**C**) The IL-6 content in the study group was significantly higher than in the control group. (**D**) The TNF-α content in the study group was significantly higher than in the control group. (**E**) The expression of BMAL1 in the study group was significantly lower than in the control group. (**F**) The expression of SIRT1 in the study group was significantly lower than in the control group. (**G**) There was a significant difference in the expression of BMAL1 and SIRT1 between the two groups. *P < 0.05.

In comparison to the control group, the study group exhibited significantly increased levels of MDA, IL-6, and TNF-α, and decreased activity of SOD (Fig. 1A–D). The relative protein expression levels of SIRT1 and BMAL1 were also compared between the two groups (Fig. 1G), with the level of BMAL1 being significantly lower in the study group than in the control group (Fig. 1E). Similarly, the level of SIRT1, which is involved in circadian clock gene transcription regulation, showed a decreasing trend in the study group (Fig. 1F).

### Comparison of MDA, SOD, IL-6, TNF-α, BMAL1, and SIRT1 among the four subgroups of the study group

**Figure 2:**
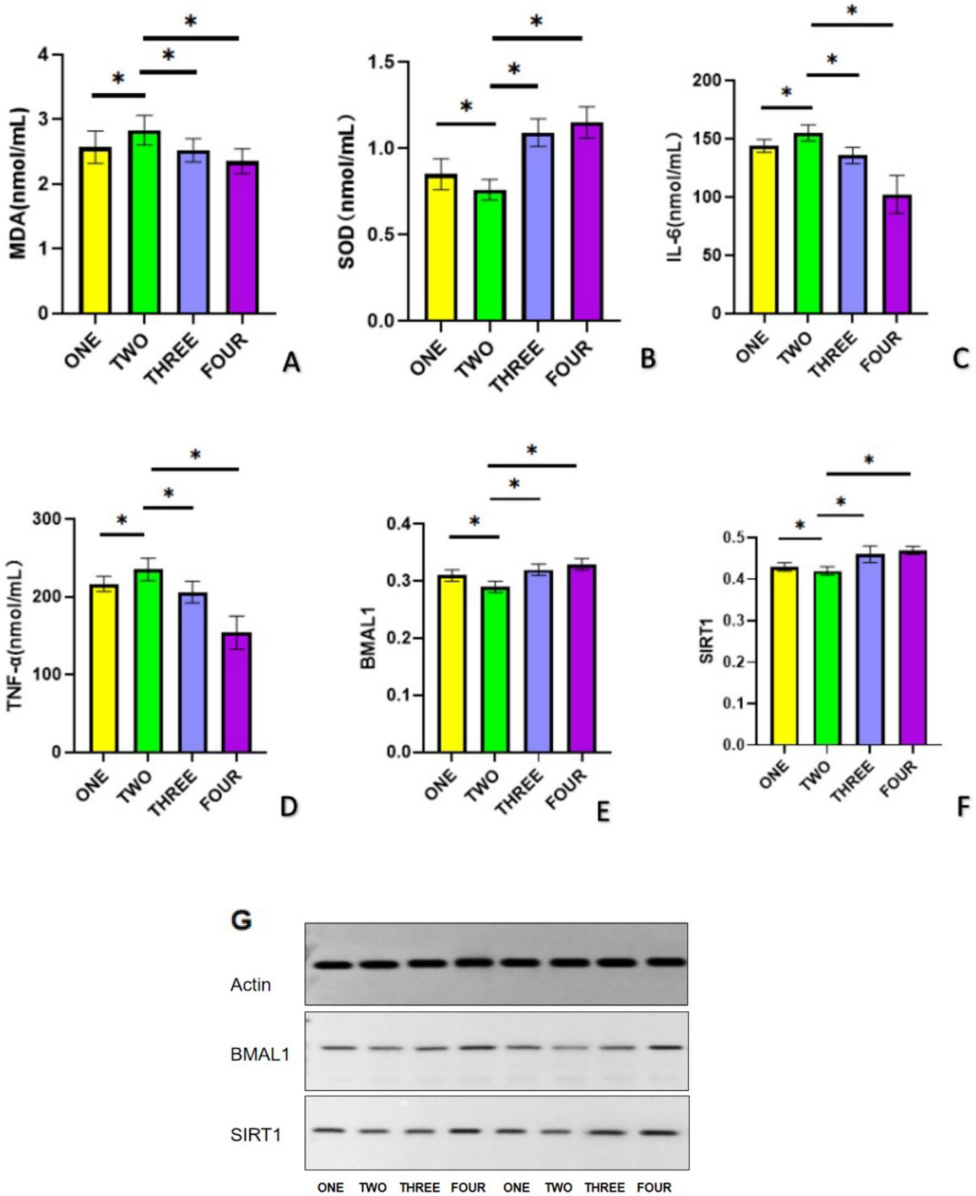
Comparison of MDA, SOD, IL-6, TNF-α, BMAL1, and SIRT1 expression in four subgroups. (**A**) There were significant differences in serum MDA concentration among the four groups (*P < 0.05); MDA content was the highest during subgroup 2 (6:00–11:59) and the lowest during subgroup 4 (18:00–23:59). (**B**) There were significant differences in serum SOD concentration among the four groups (*P < 0.05); SOD content was the lowest during subgroup 2 (6:00–11:59) and the highest during subgroup 4 (18:00–23:59). (**C**) There were significant differences in serum IL-6 concentration among the four groups (*P < 0.05); IL-6 content was the highest during subgroup 2 (6:00–11:59) and the lowest during subgroup 4 (18:00–23:59). (**D**) There were significant differences in serum TNF-α concentration among the four groups (*P < 0.05); TNF-α content was the highest during subgroup 2 (6:00–11:59) and the lowest during subgroup 4 (18:00–23:59). (**E**) The expression level of BMAL1 was different among the four groups (*P < 0.05). BMAL1 expression level was the lowest during subgroup 2 (6:00–11:59) and the highest during subgroup 4 (18:00–23:59). (**F**) The expression level of SIRT1 was different among the four groups (*P < 0.05). SIRT1 expression level was the lowest during subgroup 2 (6:00–11:59) and the highest during subgroup 4 (18:00–23:59). (**G**) Comparison of BMAL1 and SIRT1 expression in the four subgroups. *P < 0.05.

Significant circadian rhythms were observed in MDA, SOD, IL-6, TNF-α, BMAL1, and SIRT1 expression across the four subgroups of the study group (Fig. 2A–F). Specifically, patients with ischemic stroke onset from subgroup 2 (6:00–11:59) exhibited significantly increased levels of MDA, IL-6, and TNF-α, and decreased activity of SOD. Conversely, patients with onset from subgroup 4 (18:00–23:59) had the lowest levels of MDA, IL-6, and TNF-α, and the highest SOD activity. When comparing the relative expression levels of BMAL1 and SIRT1 proteins among the four subgroups (Fig. 2G), a significant decrease in BMAL1 and SIRT1 expression levels was found in patients with ischaemic stroke onset from subgroup 2 (6:00 to 11:59). There was also a trend towards higher BMAL1 and SIRT1 expression levels in patients with onset at subgroup 3 (12:00–17:59), while BMAL1 and SIRT1 expression levels peaked in patients with onset at subgroup 4 (18:00–23:59).

### Correlation analysis of MPO with BMAL1 and MDA

**Figure 3:**
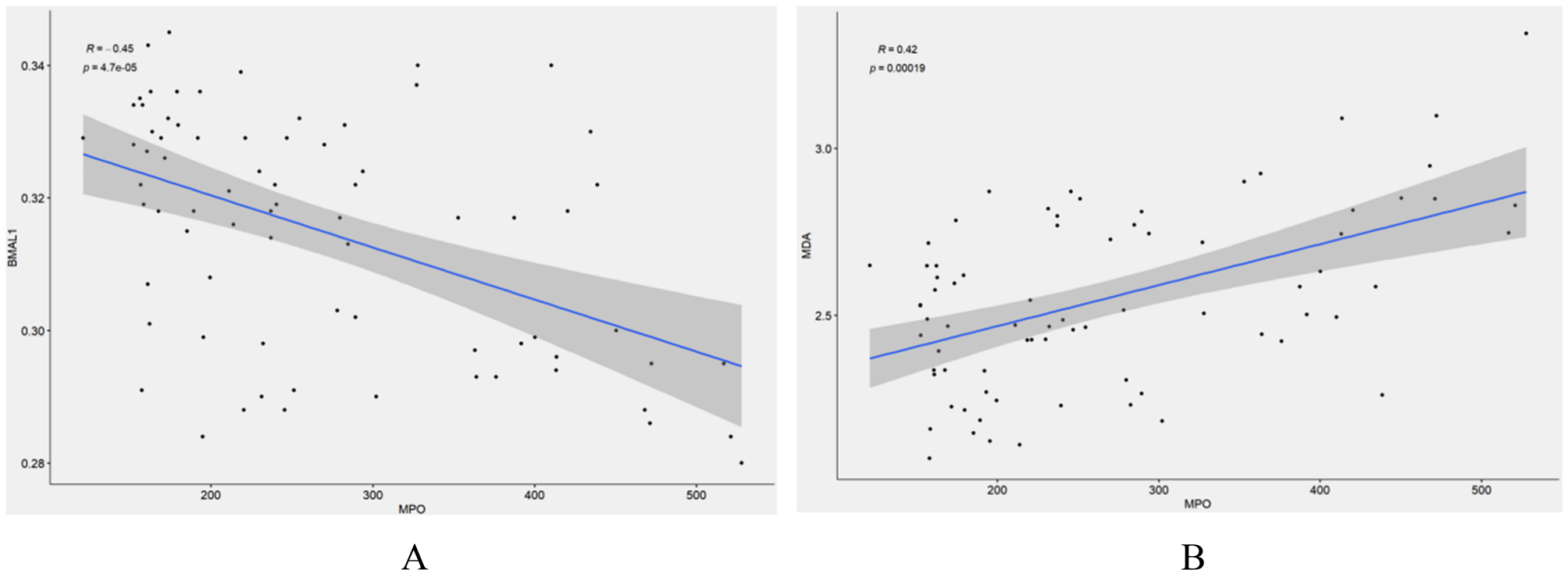
Correlation analysis between MPO, BMAL1, and MDA. (**A**) There was a moderate negative correlation between MPO and BMAL1. (**B**) There was a moderate positive correlation between MPO and MDA.

Pearson correlation analysis (Fig. 3A,B) revealed a moderate negative correlation between MPO and BMAL1 (r = -0.45), while MPO and MDA showed a moderate positive correlation (r = 0.42).

### Correlation analysis between BMAL1 and SIRT1 among the four subgroups of the study group

**Figure 4:**
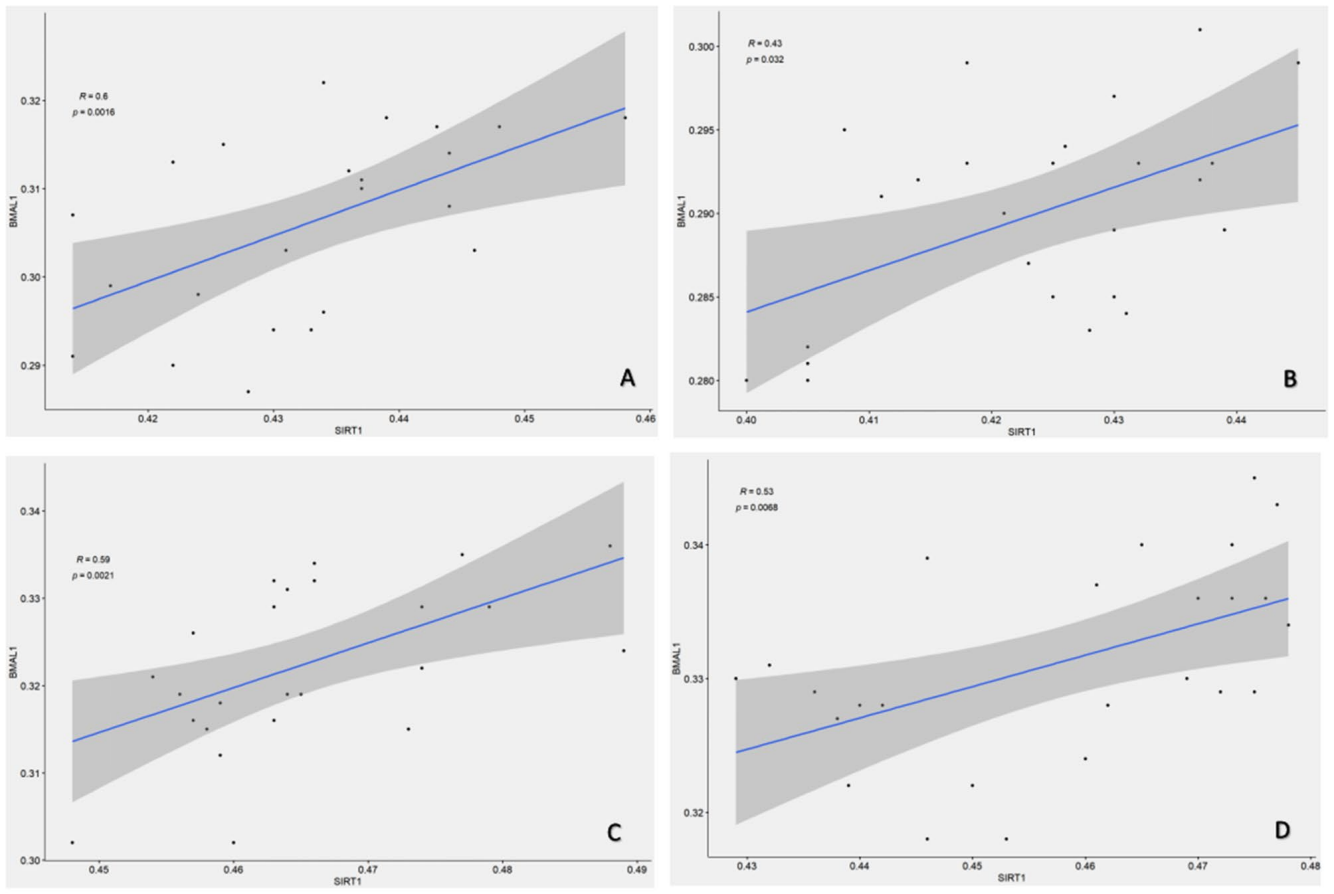
Correlation analysis between BMAL1 and SIRT1 among the four subgroups of the study group. There was a moderate positive correlation between BMAL1 and SIRT1 in (**A**) subgroup 1: 0:00–05:59. (**B**) subgroup 2: 06:00–11:59. (**C**) subgroup 3: 12:00–17:59. (**D**) subgroup 4: 18:00–23:59.

Pearson correlation analysis (Fig. 4A–D) revealed a moderate positive correlation among the four subgroups of the study group (r = 0.60, r = 0.43, r = 0.59, r = 0.53). This suggests that SIRT1-BMAL1 may play a significant role in the early pathogenesis of ischemic stroke.

#### Correlation analysis between BMAL1 and IL-6, TNF-α, SIRT1, MDA, and SOD

**Figure 5:**
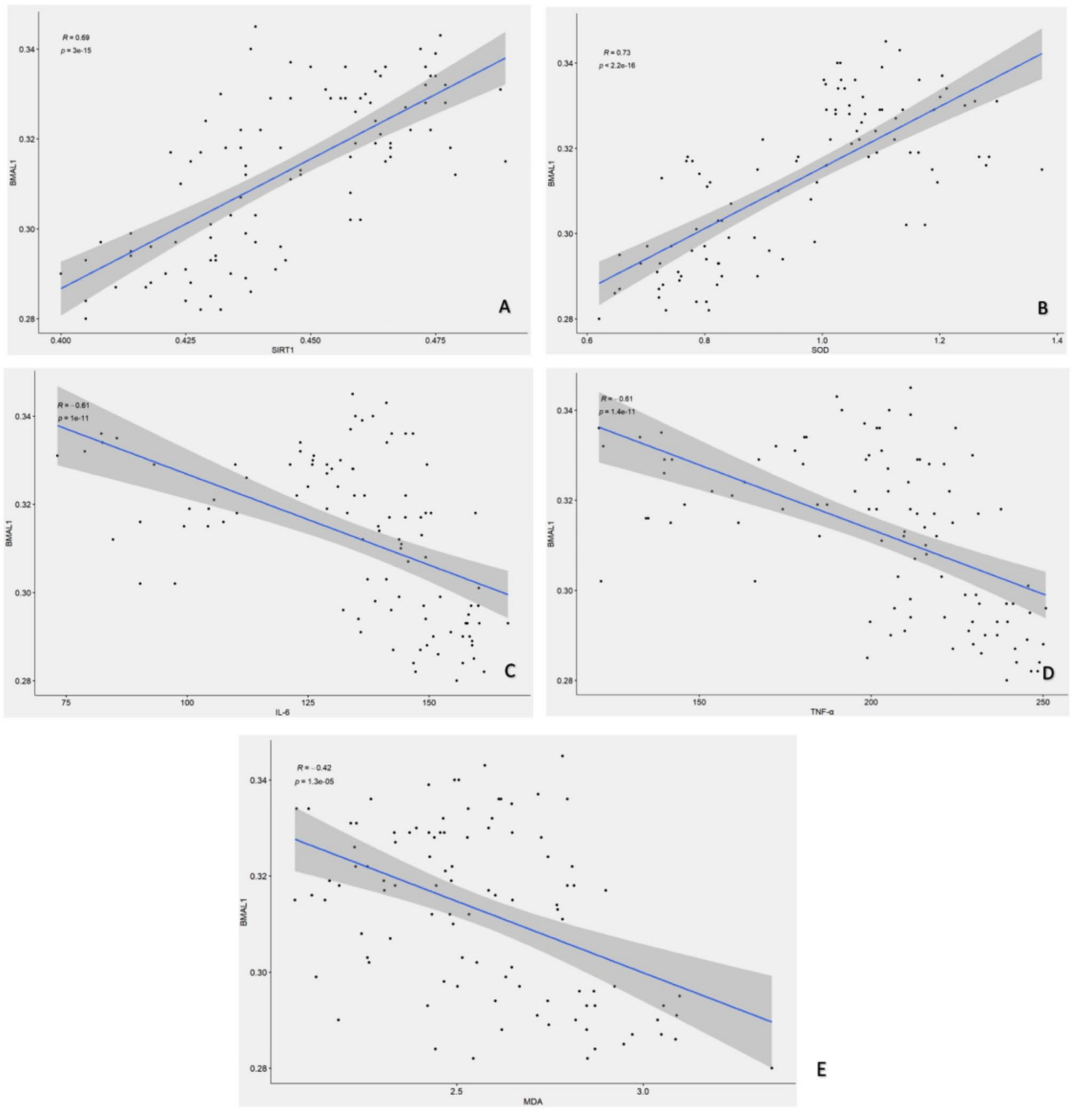
Correlation analysis between BMAL1 and SIRT1, MDA, IL-6, TNF-α, and SOD. (**A**) There was a moderate positive correlation between BMAL1 and SIRT1. (**B**) There was a moderate positive correlation between BMAL1 and SOD. (**C**, **D**) There was a moderate negative correlation between BMAL1 and both IL-6 and TNF-α. (**E**) BMAL1 showed a non-significant negative correlation with MDA.

Pearson correlation analysis (Fig. 5A–E) demonstrated a moderate positive correlation between BMAL1 and SIRT1, as well as between BMAL1 and SOD (r = 0.69; r = 0.73). In contrast, BMAL1 was moderately negatively correlated with IL-6 and TNF-ɑ (r = −0.61; r = −0.61), and also negatively correlated with MDA (r = −0.42).

## Discussion

This study aimed to detect indicators related to oxidative stress and inflammation in patients with early-onset ischaemic stroke. The results showed significantly higher levels of MDA, IL-6, TNF-α, and lower expression of SOD in the ischaemic stroke group compared to the control group, indicating the involvement of oxidative stress and inflammation in the early pathological progression of ischaemic stroke. These findings are consistent with previous studies^24^ that have also reported similar associations between oxidative stress, inflammation, and ischaemic stroke progression^25^.

Ischaemic stroke is a complex pathophysiological process involving various factors that contribute to its occurrence and progression. In the core infarct zone, rapid cellular death leads to neuronal dysfunction and loss of electrical activity and metabolism. In contrast, oxidative stress and inflammation in the peripheral ischaemic penumbra trigger cellular and molecular cascades that lead to neuronal degeneration and death^26,27^. This study found a downward trend in BMAL1 and SIRT1 expression along with increased activation of oxidative stress (MDA) and inflammatory factors (IL-6, TNF-α) in patients with early-onset ischemic stroke. As a core biological clock protein, BMAL1 plays a crucial role in regulating the circadian rhythm^19,28,29^, and previous studies have demonstrated that its transcriptional activation is regulated by acetylation/deacetylation, the circadian vibration of Per2 and BMAL1 luciferase is weakened, and transcription of BMAL1 ceases after SIRT1 deacetylation^30^. The present study found significantly lower levels of BMAL1 in the ischaemic stroke group than that in the control group, and the corresponding low expression of SIRT1 was consistent with BMAL1 oscillation, indicating that SIRT1 regulates BMAL1 and participates in the early occurrence of ischaemic stroke. Earlier studies suggested that the incidence of stroke is highest at night^31^, while more recent research found peak incidence at 06:00–12:00^32^ and bimodal pattern with peaks at 6:00–12:00 and 18:00–20:00^33^. Approximately 24% of cerebrovascular events occur within the first hour after waking^34^, and the transition from sleep to wakefulness might be an independent risk factor for stroke in the morning or after a nap^35^. This study also analyzed BMAL1 and SIRT1 levels across four different time subgroups (00:00–05:59, 06:00–11:59, 12:00–17:59, and 18:00–23:59) and found that the expression levels were lowest in patients with ischaemic stroke onset from subgroup 2 (6:00–11:59), while subgroup 3 (12:00–17:59) showed a higher trend of BMAL1 and SIRT1 levels. This suggested that the decreased expression of SIRT1-BMAL1 may be associated with a higher incidence of ischaemic cerebral infarction in the morning but not significantly associated with a higher incidence in the afternoon. Additionally, this study found that SIRT1-BMAL1 exhibited time-dependent synchronization with initial systolic blood pressure and MPO. MPO is expressed in infiltrating neutrophils, activated microglia, neurons, and astrocytes in ischemic brain tissue and catalyzes the reaction of chloride ions to produce HOCl, contributing to oxidative stress-induced modification of lipoproteins^36^, induction of endothelial dysfunction^37^, and increased plaque vulnerability^38^. Our study found MPO to be moderately positively correlated with MDA and to exhibit a circadian rhythm inversely consistent with that of SIRT1-BMAL1, suggesting that the SIRT1-BMAL1 pathway might play a certain role in the early occurrence of ischaemic stroke.

In the pathogenesis of acute myocardial infarction, BMAL1 boosts antioxidant activity by enhancing the redox state of HSPB1^39^. However, the impact of BMAL1 on oxidative stress and inflammation in the progression of ischaemic stroke remains unclear. We examined the expression levels of the antioxidant stress kinase SOD and TNF-α at different onset times of ischaemic stroke and found that their expression levels were consistent with those of SIRT1-BMAL1. Correlation analysis revealed a moderate positive correlation between BMAL1 and SIRT1 and oxidative stress factor SOD, but a negative correlation with IL-6, TNF-ɑ, and MDA. Therefore, we suggest that the SIRT1-BMAL1 pathway may involve the antioxidant response of SOD and have a negative regulatory effect on the oxidative stress factor MDA and inflammatory factors IL-6 and TNF-ɑ.

Dyslipidaemia is an independent risk factor for AIS, as demonstrated by previous studies. BMAL1 knockout in mice has been shown to cause hyperlipidemia and increase atherosclerosis^40^, likely due to BMAL1’s regulation of key lipolysis enzymes such as ATGL, LPL, and HSL^41^. However, this study did not observe significant circadian rhythm changes in TGs, TC, LDL, HDL, immediate systolic blood pressure and diastolic blood pressure, blood glucose and HCY. The unstable biological indicators during early-stage acute cerebral infarction may be due to the small he sample size of the subgroup.

In addition, the severity of ischaemic stroke at onset also showed rhythmic changes. However, unlike SIRT1-BMAL1, patients with onset at 00:00–05:59 had the highest NIHSS score while those with onset between 12:00 and 17:59 had the lowest. Shokri et al.^42^ analyzed 1450 acute ischaemic stroke patients and found that patients with morning onset had the highest NIHSS score while the lowest was observed during the period from 18:01 to 24:00. This phenomenon is likely associated with delays in seeking medical attention. Our study found that patients with onset at night had the highest NIHSS score but further analysis of related factors is needed through follow-up experiments.

This study focused on the expression of SIRT1-BMAL1 and risk factors during different onset times of ischemic stroke and revealed that SIRT1-BMAL1 may contribute to the early onset of ischemic stroke by regulating oxidative stress.

### Supplementary Information

Supplementary Information.

## Data Availability

The datasets used and/or analysed during the current study available from the corresponding author on reasonable request.
