# Supplementary material for: The role of the SIRT1-BMAL1 pathway in regulating oxidative stress in the early development of ischaemic stroke
Source: Sci Rep. 2024 Jan 20;14:1773. doi: 10.1038/s41598-024-52120-5 (PMC10799848; doi:10.1038/s41598-024-52120-5)
Supplement: Supplementary file 1 — Supplementary Information. [file 41598_2024_52120_MOESM1_ESM.pdf]

Statement

Approval Number: 2021019

In accordance with the principles of the 1964 Helsinki Declaration and its subsequent revisions, the research project titled ***The role of the SIRT1-BMAL1 pathway in regulating oxidative stress in the early development of ischaemic stroke*** has been granted approval by the Medical Ethics Committee of Taiyuan Central Hospital.

This study strictly adheres to medical ethics principles and complies with relevant national laws and regulations. The rights and autonomy of the participants are respected, and the principles of beneficence, non-maleficence, and justice are followed throughout the research process.

The research project will be conducted according to the approved protocol by the ethics committee. Informed consent procedures have been implemented, ensuring that participants or their authorized representatives fully understand the nature and purpose of the study. Measures have been taken to protect the rights, privacy, and safety of the participants.

Any modifications to the research protocol will require prior approval from the ethics committee. Regular monitoring and reporting of the progress and any adverse events will be carried out as per the approved guidelines.

We assure that this research project will be conducted with the utmost integrity and in compliance with ethical standards, ensuring the well-being and rights of the participants.

太原市中心医院医学伦理委员会

审查批件

项目名称	生物钟核心基因Bmal1蛋白基于调节炎症因子与急性缺血性脑卒中的相关性研究					
项目负责人	李伟荣	职称	主任医师	科室	神经内科	
组长单位	太原市中心医院			项目来源	山西省卫健委“四个一批”重大科技攻关专项	
研究时间	2022.1-2023.12			经费资助者	山西省卫健委	
批件编号	2021019	审查方式	☑会议审查	☐快速审查	审查日期	2021年11月24日
审查内容	课题计划任务书（研究方案）<br>知情同意书<br>初始审查申请表、主要研究者履历					
委员意见	出席	12人	投票	12人	回避	0人
	同意 9 人　作必要修改后同意 3 人　作必要修改后重审 0 人<br>不同意 0 人　暂停或终止试验 0 人					
结论	☑同意；☐作必要修改后同意；☐作必要修改后重审；☐不同意；☐暂停或终止试验					
跟踪审查频率	☐ 3个月	☐ 6个月	☐ 9个月	☑ 12个月	☐ 无	

审查意见：

经本伦理委员会审查，同意按所批准的项目方案、知情同意书开展本项目，要求研究者严格遵循医学伦理原则要求，自觉接受国家有关法律和法规约束，在项目实施中尊重受试者的自主意愿，同时遵守有益、不伤害以及公正的原则。遵循伦理委员会批准的方案开展该项目，充分落实知情同意，切实保障受试者权益和安全。

太原市中心医院医学伦理委员会

主任委员/副主任委员：

2021年11月26日

备注：请遵循伦理委员会批准的方案开展临床研究，保护受试者的健康与权利。研究过程中若变更主要研究者，对临床研究方案、知情同意书、招募材料等的任何修改，应提交修正案审查申请。发生严重不良事件，应及时提交严重不良事件报告。暂停或提前终止临床研究，应及时提交暂停或终止研究报告。研究结束后向本伦理委员会提交结题报告。

The order of load sample：

The first PVDF membrane：control group NO.48，control group NO.01，control group NO.14，control group NO.23，control group NO.70，control group NO.99，study group NO.46（Period 2 (6:00-11:59)），study group NO.04（Period 1 (0:00-05:59)），study group NO.49（Period 2 (6:00-11:59)），study group NO.52（Period 3 (12:00-17: 59)），study group NO.81（Period 4 (18:00-23:59)），study group NO.84（Period 4 (18:00-23:59)）

The second PVDF membrane：study group NO.03（Period 1 (0:00-05:59)），study group NO.42（Period 2 (6:00-11:59)），study group NO.56（Period 3 (12:00-17: 59)），study group NO.76（Period 4 (18:00-23:59)），study group NO.21（Period 1 (0:00-05:59)），study group NO.38（Period 2 (6:00-11:59)），study group NO.59（Period 3 (12:00-17: 59)），study group NO.86（Period 4 (18:00-23:59)），study group NO.19（Period 1 (0:00-05:59)），study group NO.40（Period 2 (6:00-11:59)），study group NO.69（Period 3 (12:00-17: 59)），study group NO.93（Period 4 (18:00-23:59)）

Result

1. Figures in the manuscript:

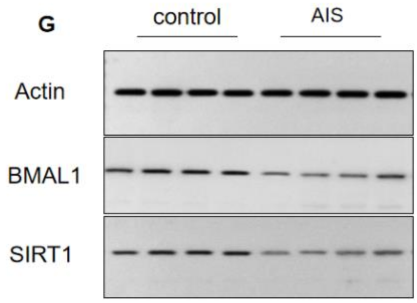

Figure 1 Comparison of MDA, SOD, IL-6, TNF-α, BMAL1 and SIRT1 between the study and control groups. (G) There was a significant difference in the expression of BMAL1 and SIRT1 between the two groups. *P<0.05.

Original picture

1.1 Beta-Actin: dilution rate 1:1000 molecular weight 43kDa

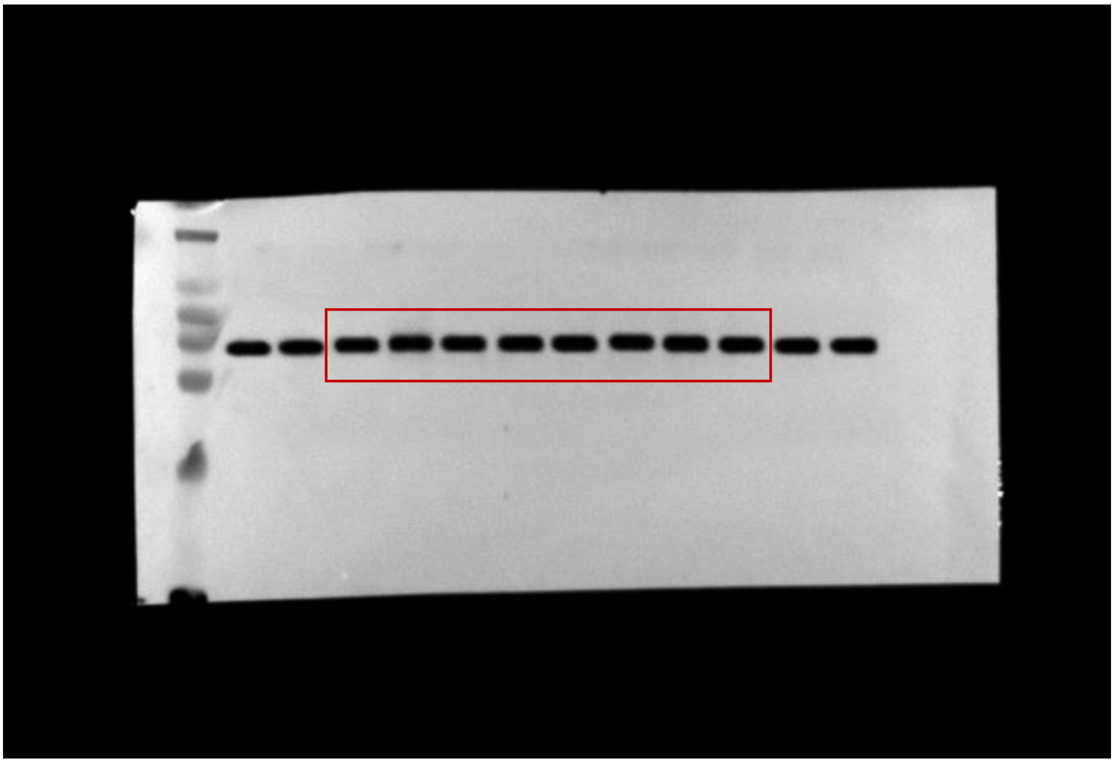

1. 2 BMAL1 **dilution rate 1:1000 molecular weight 69kDa**

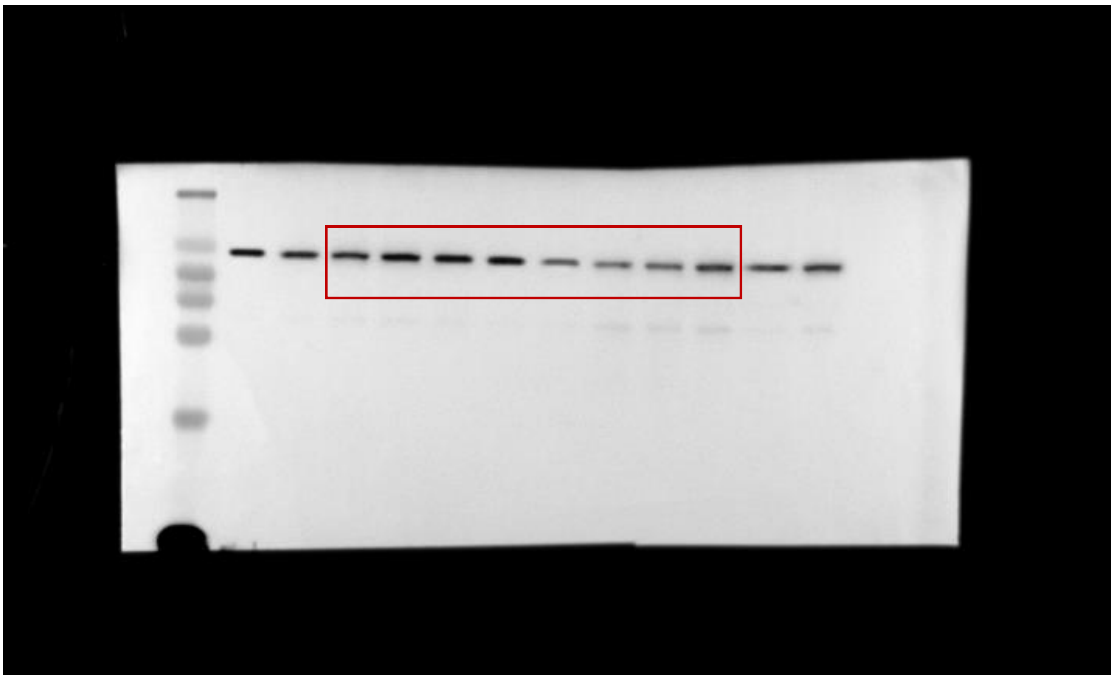

1. 3 SIRT1 **dilution rate 1:1000 molecular weight 81kDa**

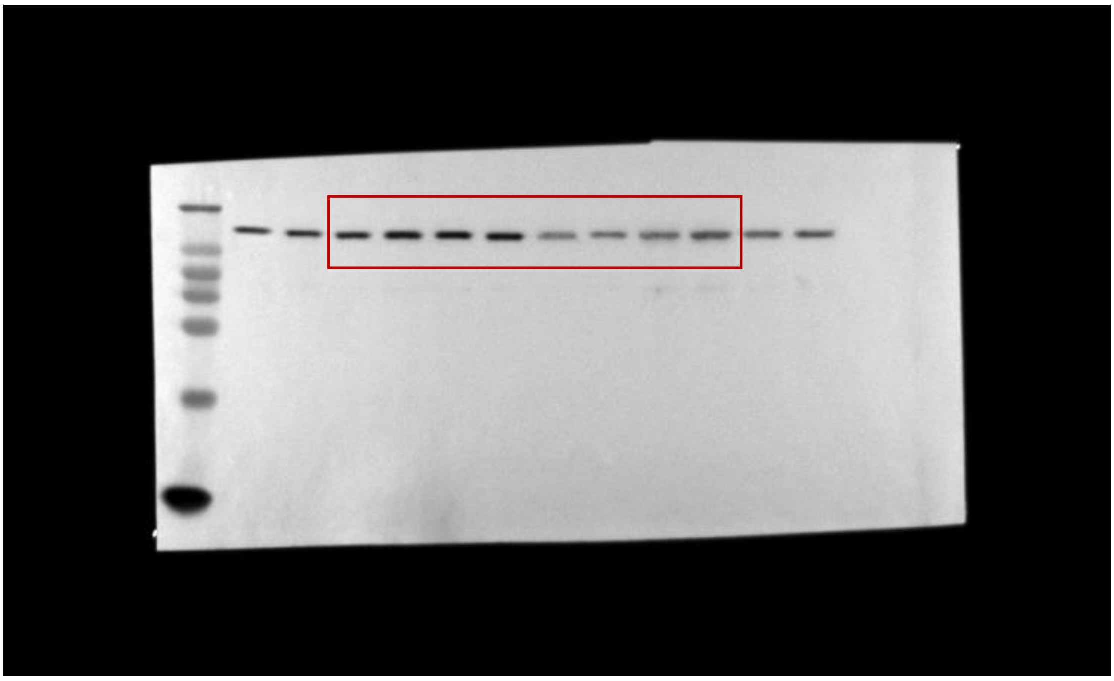

Note: The red box indicates the referenced image in the manuscript

2. Figures in the manuscript:

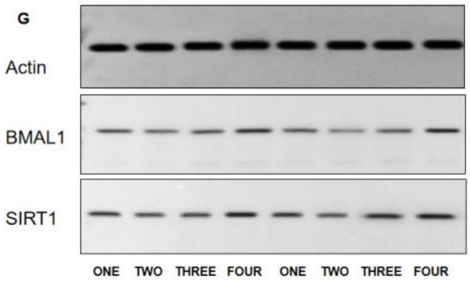

Figure 2 Comparison of MDA, SOD, IL-6, TNF-α, BMAL1, and SIRT1 expression in four subgroups. (G) Comparison of BMAL1 and SIRT1 expression in the four subgroups.

Original picture

2.1 Beta-Actin: dilution rate 1:1000 molecular weight 43kDa

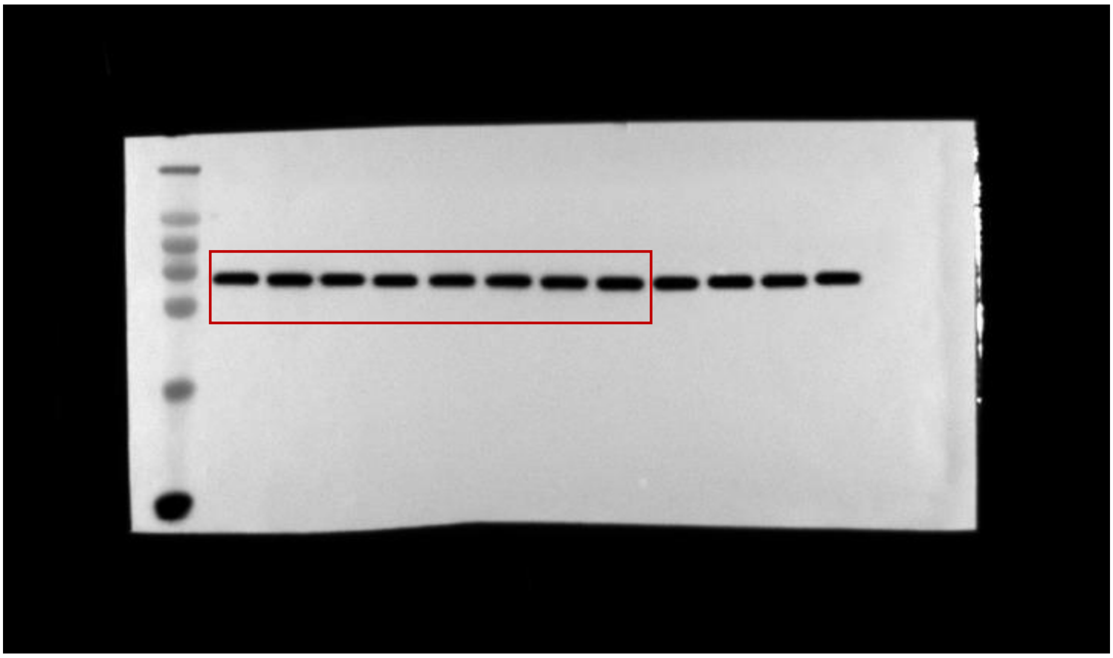

2. 2 BMAL1 **dilution rate 1:1000 molecular weight 69kDa**

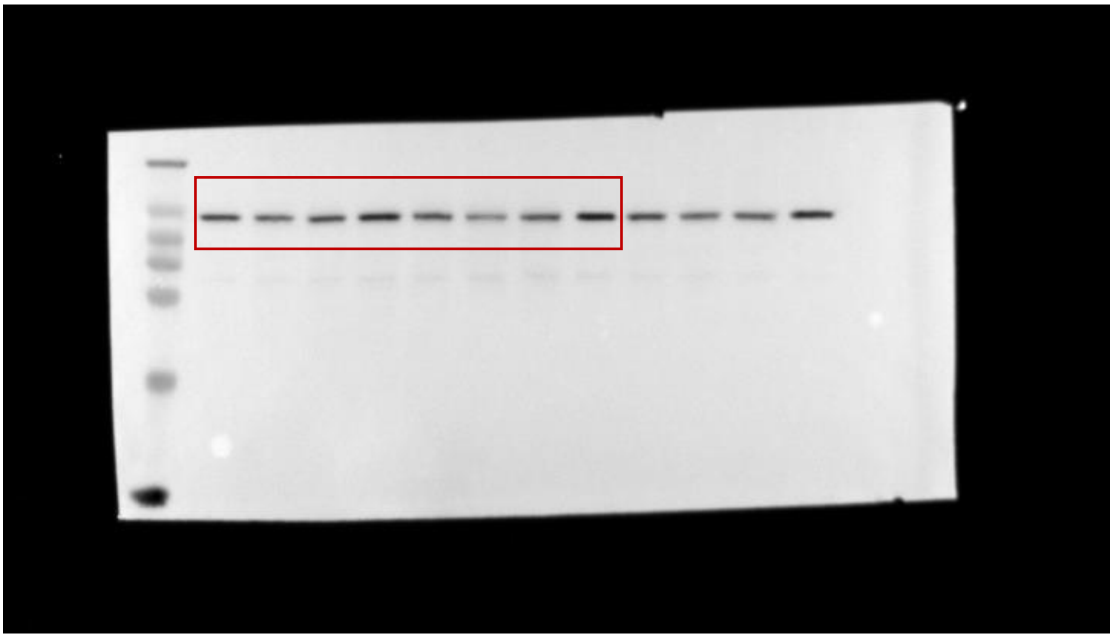

1. 3 SIRT1 **dilution rate 1:1000 molecular weight 81kDa**

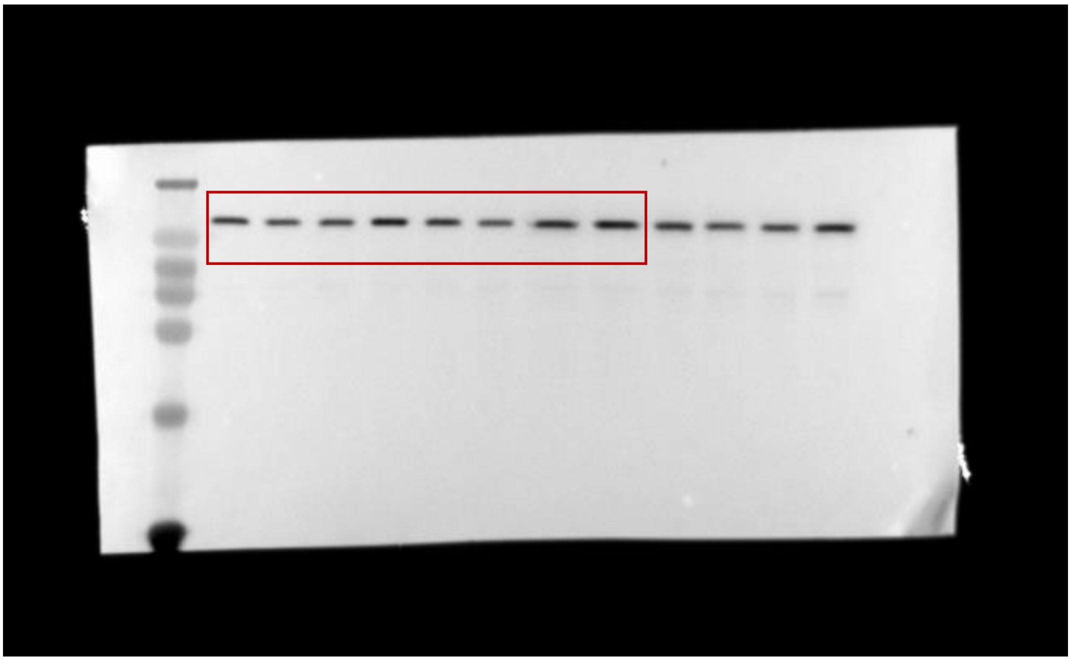

Note: The red box indicates the referenced image in the manuscript

The Relative Expression of BMAL1 and SIRT1

The first PVDF membrane	BMAL1	SIRT1
control group NO.48	0. 417391223	0. 606887692
control group NO.01	0. 404056374	0. 635972432
control group NO.14	0. 408447788	0. 621538755
control group NO.23	0. 431571389	0. 673689675
control group NO.70	0. 42292642	0. 66670915
control group NO.99	0. 426785736	0. 668927275
study group NO.46 （Period 2 (6:00-11:59)）	0. 299668464	0. 418489447
study group NO.04 （Period 1 (0:00-05:59)）	0. 287281031	0. 417313019
study group NO.49 （Period 2 (6:00-11:59)）	0. 280241191	0. 404861202
study group NO.52 （Period 3 (12:00-17: 59)）	0. 329507246	0. 454972255
study group NO.81 （Period 4 (18:00-23:59)）	0. 324405927	0. 429478592
study group NO.84 （Period 4 (18:00-23:59)）	0. 322262556	0. 440124605

The second PVDF membrane	BMAL1	SIRT1
study group NO.03 （Period 1 (0:00-05:59)）	0. 308292778	0. 457811838
study group NO.42 （Period 2 (6:00-11:59)）	0. 28856724	0. 432387558
study group NO.56 （Period 3 (12:00-17: 59)）	0. 301898876	0. 447756431
study group NO.76 （Period 4 (18:00-23:59)）	0. 317867584	0. 478196534
study group NO.21 （Period 1 (0:00-05:59)）	0. 31239592	0. 448416364
study group NO.38 （Period 2 (6:00-11:59)）	0. 279972571	0. 430121199
study group NO.59 （Period 3 (12:00-17: 59)）	0. 301681536	0. 459525674
study group NO.86 （Period 4 (18:00-23:59)）	0. 333879268	0. 475286211
study group NO.19 （Period 1 (0:00-05:59)）	0. 312660129	0. 447656767
study group NO.40 （Period 2 (6:00-11:59)）	0. 286427105	0. 439463967
study group NO.69 （Period 3 (12:00-17: 59)）	0. 315628387	0. 457834231
study group NO.93 （Period 4 (18:00-23:59)）	0. 326523277	0. 46899289

The Gray value of WB strip

The second PVDF	Beta-Actin	BMAL1	SIRT1
study group NO.03（Period 1 (0:00-05:59)）	93313	38768	42720
study group NO.42（Period 2 (6:00-11:59)）	88870	37094	53934
study group NO.56（Period 3 (12:00-17: 59)）	91551	27639	40993
study group NO.76（Period 4 (18:00-23:59)）	91643	29130	43823
study group NO.21（Period 1 (0:00-05:59)）	[illegible]	[illegible]	[illegible]
study group NO.38（Period 2 (6:00-11:59)）	94060	26334	40457
study group NO.46（Period 2 (6:00-11:59)）	90346	27074	37809
study group NO.04（Period 1 (0:00-05:59)）	89031	35577	37154

The first PVDF	Beta-Actin	BMAL1	SIRT1
control group NO.48	103243	30370	45506
control group NO.01	94518	38191	60111
control group NO.14	89503	36557	55630
control group NO.33	97829	42220	65906
control group NO.70	[illegible]	[illegible]	[illegible]
control group NO.39	89721	38291	60017
study group NO.59（Period 3 (12:00-17: 59)）	96419	29088	44307
study group NO.86（Period 4 (18:00-23:59)）	98864	33009	46989
study group NO.49（Period 2 (6:00-11:59)）	93305	26148	37776
study group NO.52（Period 3 (12:00-17: 59)）	91489	30146	41625
study group NO.81（Period 4 (18:00-23:59)）	90563	29379	38895
study group NO.84（Period 4 (18:00-23:59)）	91981	29642	40483

study group NO.19<br>（Period 1 (0:00-05:59)）	89225	27897	39942
study group NO.40<br>（Period 2 (6:00-11:59)）	98964	28346	43491
study group NO.69<br>（Period 3 (12:00-17: 59)）	91929	29015	42088
study group NO.93<br>（Period 4 (18:00-23:59)）	94512	30860	44325
